# Polyurethane nanofibrous scaffolds for wound repair: from electrospinning fabrication to biological performance evaluation

**DOI:** 10.1186/s12896-026-01114-4

**Published:** 2026-03-11

**Authors:** A. Shaker, Sara K. Tawfiq, Amal E. Alshorbagy, Mohamed T. Selim

**Affiliations:** 1 Mechanical Design and Production Engineering Department, Zagazig University, Zagazig, 44519 Egypt; 2 Nanomaterials Lab, Zagazig University, Zagazig, 44519 Egypt; 3 Mechatronics Engineering Department, Delta University for Science and Technology, Mansoura, 11152 Egypt; 4 Botany and Microbiology Department, Faculty of Science, Al-Azhar University, Cairo, 11884 Egypt

**Keywords:** Wound healing, Olive oil, Polyurethane, Electrospinning, Nanofibers, Copper oxide

## Abstract

This study presents the electrospinning fabrication of thermoplastic polyurethane (TPU) nanofibrous scaffolds with two fiber architectures; randomly oriented and unidirectionally aligned incorporating olive oil and copper oxide nanoparticles (CuO NPs) to enhance biomedical performance. Both scaffold types exhibited robust antibacterial activity: randomly oriented TPU/CuO mats produced inhibition zones of 33.0 mm against Staphylococcus aureus and 19.46 mm against *Escherichia coli*, whereas aligned mats showed a slight reduction to a 31.0 mm zone against *S. aureus.* Cytocompatibility assays using HFB4 fibroblasts demonstrated low cytotoxicity, with cell viability rates of 86% for randomly oriented fibers and 96.25% for aligned fibers. Whole-blood clotting tests confirmed rapid hemostatic action in both orientations, underscoring their capacity to promote blood coagulation. Collectively, these multifunctional TPU/CuO/olive oil nanofibrous membranes combine controlled fiber orientation with bioactive filler incorporation to deliver enhanced antimicrobial efficacy, superior cell compatibility, and effective hemostatic properties that position them as promising candidates for next-generation wound-healing dressings.

**Supplementary Information:**

The online version contains supplementary material available at 10.1186/s12896-026-01114-4.

## Introduction

The electrospinning technique has enabled the rapid fabrication of nanofibers that have been extensively applied in biomedical fields, including wound healing [1, 2], tissue scaffolding [3], drug delivery [3–5], and biosensors [6]. More recently, efforts have been centered on designing porous nanofiber dressings with high surface area and dermis-mimetic architecture to promote wound healing by mitigating infection and inflammation.

Accelerating the wound healing process remains a critical clinical challenge, as impaired repair can give rise to serious complications. Treatment strategies depend on the wound’s size, depth, and severity. Superficial injuries confined to the epidermis and papillary dermis typically heal by regenerating residual hair follicles and other epidermal appendages, whereas chronic wounds almost invariably extend through the full skin thickness [7]. An optimal wound dressing must be cost-effective and promote patient comfort throughout recovery [8]. Such dressings are engineered to maintain a moist environment, absorb excess exudate, minimize further tissue trauma, and act as a barrier against external microbes [8, 9].

Several polymers are fundamental to the design of wound healing dressings, imparting flexibility, moisture retention, and antibacterial functionality. Polyurethane (PU), polyethylene glycol (PEG), and polylactic acid (PLA) are among the most widely adopted materials due to their favorable biomedical profiles. Thermoplastic polyurethane (TPU) has garnered particular interest for its versatility and extensive industrial applications [10, 11]. Unlike many conventional polymers, TPU possesses mechanical and physical properties that closely emulate those of human cells. Its low platelet adhesion and minimal in vitro protein adsorption further enhance its biocompatibility, making TPU an outstanding candidate for advanced wound care solutions [12].

Olive oil has attracted increasing interest in biomedical and wound-healing applications due to its rich content of bioactive compounds, including phenolics, tocopherols, and unsaturated fatty acids, which are known to exhibit antioxidants, anti-inflammatory, and skin-regenerative properties [13]. In the context of polymeric scaffolds, olive oil has been reported to play an important role in improving fiber morphology, surface wettability, and cellular responses, particularly fibroblast adhesion and proliferation, which are critical for effective wound healing [13]. While olive oil may exhibit mild antimicrobial activity depending on its composition, this effect is generally limited and highly variable, and it should not be considered a primary antibacterial agent when compared to inorganic nanoparticles [14, 15]. Therefore, in the present study, olive oil was incorporated into all TPU-based formulations primarily as a bioactive functional additive to enhance processing stability, biocompatibility, and tissue interaction, rather than as an independent antimicrobial variable. Consequently, the antibacterial performance observed in this work is mainly attributed to the presence of CuO nanoparticles, while the contribution of olive oil is discussed in terms of its supportive role in scaffold morphology and cytocompatibility.

Incorporating potent antibacterial and anti-inflammatory agents - such as olive oil, copper oxide nanoparticles (CuO NPs), silver nanoparticles (Ag NPs), and zinc oxide nanoparticles (ZnO NPs) - into wound dressings significantly enhances their therapeutic performance. Copper oxide (CuO) is especially valued for its exceptional chemical stability, broad‐spectrum antimicrobial efficacy, and low cytotoxicity [16]. Beyond its bactericidal action, CuO also supports critical physiological processes including endothelial cell proliferation, angiogenesis, and stabilization of skin proteins, thereby promoting tissue repair [17]. When engineered at the nanoscale, CuO exhibits rapid and potent antimicrobial activity, eradicating over 99.9% of common bacterial pathogens within two hours at effective concentrations, which underscores its promise for clinical wound‐care applications [18].

Electrospun nanofibers, with their high porosity, extracellular matrix–mimetic architecture, and large surface area for functionalization, have become indispensable in advanced wound dressings, enabling controlled moisture balance, efficient exudate management, barrier protection, and localized delivery of therapeutic agents to accelerate tissue regeneration [19, 20].

For instance, Hong et al. [21] fabricated antibacterial polyurethane nanofiber mats by incorporating silver nanoparticles into a polycarbonate diol/isosorbide matrix via electrospinning. The resulting mats exhibited excellent flexibility and demonstrated robust activity against *Staphylococcus aureus* and methicillin-resistant *S. aureus* (MRSA). In vitro cytocompatibility assays using the immortalized human keratinocyte HaCaT cell line confirmed that both the pristine and silver-loaded polyurethane textiles were non-toxic. Fouda et al. [22] employed electrospinning to produce nanofiber mats composed of carboxymethyl chitosan (CMCTS) blended with polyethylene oxide (PEO) and integrated with silver nanoparticles. These composite mats showed potent antimicrobial efficacy against a range of pathogenic and non-pathogenic bacteria, as well as the fungus *Candida albicans* ATCC 90,028.

Unnithan et al. [23] employed electrospinning to fabricate a ternary nanofiber mat composed of dextran, polyurethane (PU), and ciprofloxacin HCl, producing a defect-free structure with highly uniform fiber diameters. SEM, FTIR, and thermal analyses confirmed homogeneous drug distribution and preserved scaffold integrity. Antimicrobial assays demonstrated approximately 99% inhibition of representative Gram-positive and Gram-negative strains.

Tan et al. [24] developed “smart” composite nanofiber mats (CNMs) by electrospinning a blend of chitosan, gelatin, and shape-memory polyurethane (SMPU), followed by a silver-nitrate post-treatment to anchor Ag⁺ onto the fiber surfaces. These CNMs exhibited programmable shape-memory behavior, optimal water-vapor transmission rates, and hydrophilic surfaces, while delivering broad-spectrum antibacterial activity against both Gram-negative and Gram-positive strains. Furthermore, they demonstrated significant fibroblast cytocompatibility and rapid hemostasis in whole-blood clotting assays.

To date, only a handful of studies have directly evaluated how the orientation of electrospun nanofibers influences antimicrobial efficacy in wound dressings, leaving a critical gap in our understanding of how fibre alignment can be harnessed to optimize infection control. Rezvani Ghomi et al. [25] demonstrated that both randomly oriented and highly aligned PCL/gelatin mats loaded with ε-polylysine showed equally strong activity against MRSA, *S. aureus*,* E. coli*, *A. baumannii*, and *P. aeruginosa*, while alignment significantly enhanced tensile strength and guided fibroblast migration without diminishing antimicrobial efficacy.

Ghobril and Grøndahl [26] reviewed that fiber alignment does not compromise the ability of nanofibrous dressings to incorporate and steadily release antibacterial agents; rather, aligned architectures can improve mechanical durability and directional fluid transport, supporting sustained antimicrobial action under dynamic wound conditions.

A comparative study on chitosan/PVA electrospun mats showed that aligned fibers achieved over 95% inhibition of *S. aureus* and *E. coli*, comparable to random mats, while offering improved strength and controlled fluid uptake that supports sustained antibacterial activity at the wound interface [27].

This study reports the electrospinning of thermoplastic polyurethane (TPU) nanofibrous membranes and provides a comprehensive evaluation of their morphological and biological properties for wound‑healing applications. To elucidate the role of fiber orientation, both randomly oriented and aligned TPU/CuO mats were fabricated and systematically characterized. Although notable progress has been achieved in nanofiber fabrication, the combined influence of fiber alignment on membrane integrity and biomedical performance remains insufficiently explored. Accordingly, this work investigates how fiber orientation modulates membrane morphology, antimicrobial activity, homeostatic behavior, and cytocompatibility. Unlike earlier studies that concentrated mainly on the biological effects of electrospun dressing components and polymer blends, the present work integrates both compositional and orientation aspects. Copper oxide nanoparticles at varying concentrations, together with olive oil, were incorporated into a thermoplastic polyurethane (TPU) matrix under systematic control of fiber orientation. This integrated approach yields a multifunctional wound dressing platform that unites structural guidance with antibacterial and antioxidant properties.

## Materials and methods

### Materials

Thermoplastic polyurethane (TPU-S80A) supplied with a number-average molecular weight (M_n_) of about 15,000 g/mol was sourced from Elastollan Co., Ltd. (Japan). N, N-Dimethylformamide (DMF; Alpha Chemika, India) served as the solvent for preparing the polymer solution. Copper oxide (CuO, 99% purity) was supplied by Nano Gate Co., Ltd. (Egypt), and 100% pure Egyptian ultra-virgin olive oil was obtained from a local market in Sinai. Pure cultures of *Staphylococcus aureus* ATCC 6538, *Bacillus subtilis* ATCC 6633, *Escherichia coli* ATCC 11,229, *Aspergillus brasiliensis* ATCC 16,404, and *Klebsiella pneumoniae* ATCC 4352 were acquired from Sigma-Aldrich Co., Ltd. (USA). Normal cell line melanocytes (HFB4) were procured from Vacsera Co. (Egypt).

### Electrospinning of TPU and TPU/CuO mats

Four distinct nanofibrous scaffolds, pure TPU and TPU/CuO/olive-oil composites, were fabricated in both random and unidirectional orientations via electrospinning. For the pure TPU system, TPU-S80A pellets were dissolved in DMF at 10 wt% and stirred magnetically overnight at room temperature. Composite solutions were prepared by first dispersing CuO nanoparticles (1, 2, and 3 wt%) in DMF using an ultrasonic processor (Hielscher UP200S, Germany), then adding TPU to reach 10 wt%. To incorporate olive oil, 5 wt% Ultra-virgin Egyptian olive oil was introduced into the TPU/CuO suspension and stirred for one hour. The final spinning solution for composite membranes contained 10 wt% TPU, 3 wt% CuO, and 5 wt% olive oil.

The optimal concentration of olive oil for electrospinning was established at 5 wt% to achieve a balance between structural integrity and bioactivity. Previous studies have shown that incorporating essential oils at levels below 3 wt% often results in bead formation and poor fiber morphology [28], whereas concentrations exceeding 5 wt% lead to phase separation and instability within the polymer matrix [29]. Increasing oil content typically elevates solution viscosity while reducing surface tension and conductivity, thereby impairing the stretching of the electrified jet during electrospinning and producing thicker, less uniform fibers [28–30]. Accordingly, 5 wt% was selected as the optimal loading, as it enables smooth fiber formation, uniform dispersion, and retention of functional properties [16, 29].

**Fig. 1:**
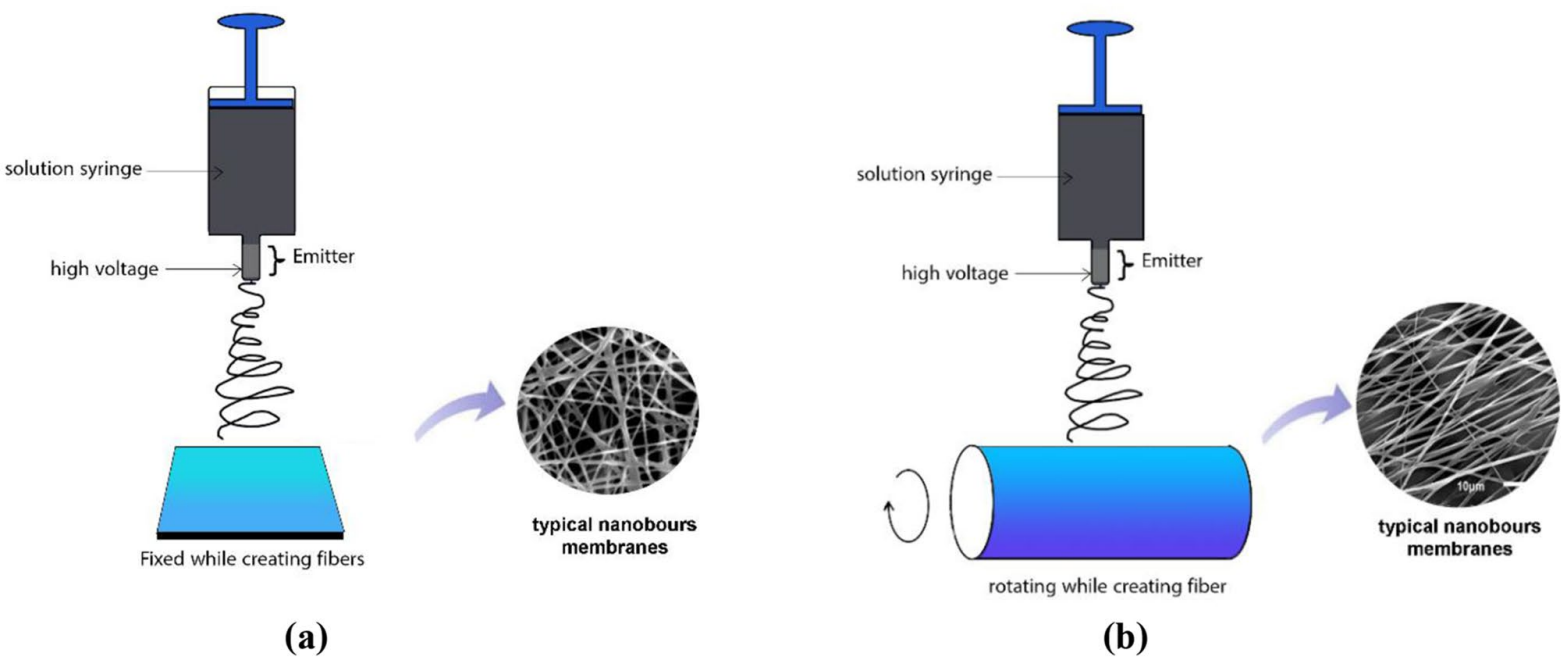
Schematic diagrams of electrospinning of (**a**) random and (**b**) aligned nanofibers

Each solution was loaded into a 5 mL syringe and electrospun at room temperature with NANON-01B apparatus (MECC Co., Ltd., Japan) fitted with a dehumidifier to maintain low-humidity conditions and minimize bead formation. Random fibers were collected on a stationary flat plate, while aligned fibers were drawn onto a rotating drum collector as shown in Fig. 1. Key parameters including applied voltage, flow rate, tip-to-collector distance, and ambient humidity, were systematically varied and optimized. Optimal settings for pure TPU mats were 25 kV voltage and 0.76 mL/h feed rate, whereas TPU/CuO composites required 29 kV voltage and 0.90 mL/h feed rate under identical orientation protocols.

The variation in applied voltage was intentional and derived from preliminary optimization experiments. Pure TPU solutions exhibited stable jet formation and uniform fiber deposition at 25 kV. In contrast, the incorporation of CuO nanoparticles and olive oil altered the solution’s conductivity and viscosity, necessitating a slightly higher voltage (29 kV) to sustain continuous jetting and minimize bead formation. This adjustment was essential to preserve stable electrospinning conditions across both sample groups. As consistently reported in the literature, solution properties such as conductivity and viscosity exert a critical influence on electrospinning stability and fiber morphology [31, 32]. Accordingly, the applied voltage was carefully tailored to the specific characteristics of each solution, ensuring uniform fiber formation in both TPU and TPU/CuO systems.

To fabricate unidirectionally oriented nanofibrous membranes, a rotating drum collector was used operating at 1500 rpm for pure TPU and 1600 rpm for TPU/CuO mats. A fixed tip-to-collector distance of 150 mm was maintained throughout all runs. The adjustment in collector rotation speed was made to account for the influence of CuO nanoparticles and olive oil on fiber solidification and alignment. For pure TPU solutions, a speed of 1500 rpm was sufficient to produce well‑aligned fibers with consistent morphology. In contrast, the incorporation of CuO nanoparticles and olive oil altered the viscoelastic properties of the composite solution, necessitating a slightly higher speed (1600 rpm) to enhance fiber stretching and orientation. This minor modification did not introduce significant differences in fiber morphology between the groups; rather, it ensured comparable alignment and uniformity across samples. Previous studies have demonstrated that collector rotation speed plays a critical role in determining fiber orientation and morphology [33–35]. In line with these findings, our adjustment was carefully tailored to the specific solution characteristics, thereby maintaining consistency in fiber quality across both TPU and TPU/CuO samples. Electrospinning was carried out under controlled ambient conditions with relative humidity held at 20 ± 3%. It is worth mentioning that both random and aligned samples were electrospun during the same session under identical conditions to minimize batch effects.

### Characterization

Morphological and elemental analyses were conducted by scanning electron microscopy (SEM; JEOL JSM-6510 IV, Japan) at 20 kV, coupled with an energy-dispersive X-ray spectrometer (EDX) for elemental mapping. Chemical bonding and functional groups were identified via Fourier transform infrared spectroscopy (FTIR; Nicolet iS10, Thermo Scientific, UK) across the 650–4000 cm⁻¹ region at ambient temperature.

### Antimicrobial assessment

Agar disc diffusion (Kirby–Bauer) assays were employed to evaluate the antimicrobial efficacy of the nanofibrous membranes [36, 37]. Pure cultures of *Bacillus subtilis* ATCC 6633, *Staphylococcus aureus* ATCC 6538, *Escherichia coli* ATCC 11,229, and *Klebsiella pneumoniae* ATCC 4352 were revived in Mueller–Hinton broth, while *Aspergillus brasiliensis* ATCC 16,404 was cultured in potato dextrose agar (PDA). The bacterial inocula were adjusted to a turbidity equivalent to the 0.5 McFarland standard (approximately 10⁶ CFU/mL). Sterile Mueller–Hinton agar plates were uniformly inoculated by evenly spreading the microbial suspensions using a sterile cotton swab. Square membrane specimens (1 cm × 1 cm) of randomly oriented and unidirectionally aligned fibers, including pure TPU and TPU/CuO nanocomposites containing 1, 2, and 3 wt% CuO nanoparticles, were aseptically placed onto the surface of the inoculated agar plates. To facilitate the diffusion of active components from the membranes into the agar medium, the plates were initially maintained at 4 °C for 1 h. Subsequently, bacterial plates were incubated at 37 °C for 24 h under aerobic conditions, without CO₂ supplementation. Fungal plates inoculated with *A. brasiliensis* were incubated at 28 °C for 72 h on PDA medium. Following incubation, the diameters of the inhibition zones surrounding each membrane were measured in millimeters using a calibrated ruler. All experiments were performed in triplicate, and the results were expressed as mean inhibition zone diameters ± standard deviation. The Kirby–Bauer method was selected due to its low cost, operational simplicity, reproducibility, and broad applicability across a wide range of microbial species, enabling straightforward interpretation of antimicrobial activity [37].

### Whole blood clotting

Whole blood clotting was evaluated according to previously described methods [38, 39]. Citrated blood was prepared by mixing whole blood with citrate–dextrose anticoagulant (containing 5 mM d-glucose) at a 9:1 ratio. Triplicate specimens (1.0 × 1.0 cm²) of both randomly oriented and aligned pure TPU membranes, as well as TPU/CuO mats containing 1, 2, and 3 wt% CuO NPs were placed individually in 15 mL plastic Petri dishes. Citrate blood (0.1 mL) was applied to each sample surface, and coagulation was triggered by adding 10 µL of 0.2 M CaCl₂. After incubation at 37 °C for 15 min, red blood cells not incorporated into the clot were lysed by adding 10 mL of deionized water, and the absorbance of the released hemoglobin was measured at 540 nm. The absorbance of 0.1 mL of whole blood diluted in 10 mL of deionized water served as the reference.

### In vitro cytotoxicity of TPU/CuO mats

The cytotoxicity of TPU/CuO membranes was evaluated using the MTT assay (3-(4,5-dimethylthiazol-2-yl)-2,5-diphenylterazolium bromide) on HFB4 normal human fibroblast cell lines (nh-skip-FB0040) at Science Way for Research and Scientific Consultations (Cairo, Egypt), to assess their suitability as antimicrobial wound-healing dressings. Randomly oriented and unidirectionally aligned TPU/CuO membranes containing 3 wt% CuO nanoparticles were punched into disks, sterilized under UV light for 20 min, and individually placed in 24-well tissue culture plates. Each well was seeded with HFB4 cells at a density of 1 × 10⁵ cells/mL (100 µL) and incubated at 37 °C for 24 h to allow the formation of a confluent monolayer. Untreated cells were used as controls, and all experiments were performed in triplicate. After incubation, cell morphology was examined microscopically to detect any cytotoxic effects, including cell rounding, monolayer disruption, shrinkage, or detachment. Subsequently, 50 µL of MTT solution was added to each well, and the plates were gently agitated at 150 rpm for 5 min to ensure uniform distribution. The plates were then incubated at 37 °C for 24 h in a humidified atmosphere containing 5% CO₂ to allow for formazan crystal formation. The MTT solution was carefully removed, and 200 µL of 10% dimethyl sulfoxide (DMSO) was added to each well to dissolve the formazan crystals. After incubation for 30 min, the absorbance was measured at 560 nm using an ELISA microplate reader. Cell viability was calculated relative to untreated controls according to Eq. (1):

1 $$\:\mathrm{C}\mathrm{e}\mathrm{l}\mathrm{l}\:\mathrm{v}\mathrm{i}\mathrm{a}\mathrm{b}\mathrm{i}\mathrm{l}\mathrm{i}\mathrm{t}\mathrm{y}\:\left(\mathrm{\%}\right)=\:\left[\frac{\mathrm{A}\mathrm{b}\mathrm{s}\mathrm{o}\mathrm{r}\mathrm{b}\mathrm{a}\mathrm{n}\mathrm{c}\mathrm{e}\:\mathrm{o}\mathrm{f}\:\mathrm{t}\mathrm{r}\mathrm{e}\mathrm{a}\mathrm{t}\mathrm{m}\mathrm{e}\mathrm{n}\mathrm{t}}{\mathrm{A}\mathrm{b}\mathrm{s}\mathrm{o}\mathrm{r}\mathrm{b}\mathrm{a}\mathrm{n}\mathrm{c}\mathrm{e}\:\mathrm{o}\mathrm{f}\:\mathrm{c}\mathrm{o}\mathrm{n}\mathrm{t}\mathrm{r}\mathrm{o}\mathrm{l}}\right]\mathrm{*}\:100$$

The same MTT protocol was applied for evaluating the cytotoxicity of both randomly oriented and aligned TPU/CuO membranes.

### Statistical analysis

Statistical analyses were performed in Minitab^®^ version 18 (2017), with results expressed as mean ± standard deviation. Group differences were evaluated by one-way analysis of variance (ANOVA), followed by Tukey’s post-hoc test for pairwise comparisons as previously described [40]. A p-value below 0.05 was considered statistically significant.

### Ethical approval

The consent form and the research proposal were approved, and the research was conducted by the principles of the Declaration of Helsinki. Additionally, the experimental study was authorized by the ethics committee of the Faculty of Science at Al-Azhar University, Assiut, Egypt, with a Certificate Reference Number: AZHAR 7/2024.

## Results and discussion

### Morphology

**Fig. 2:**
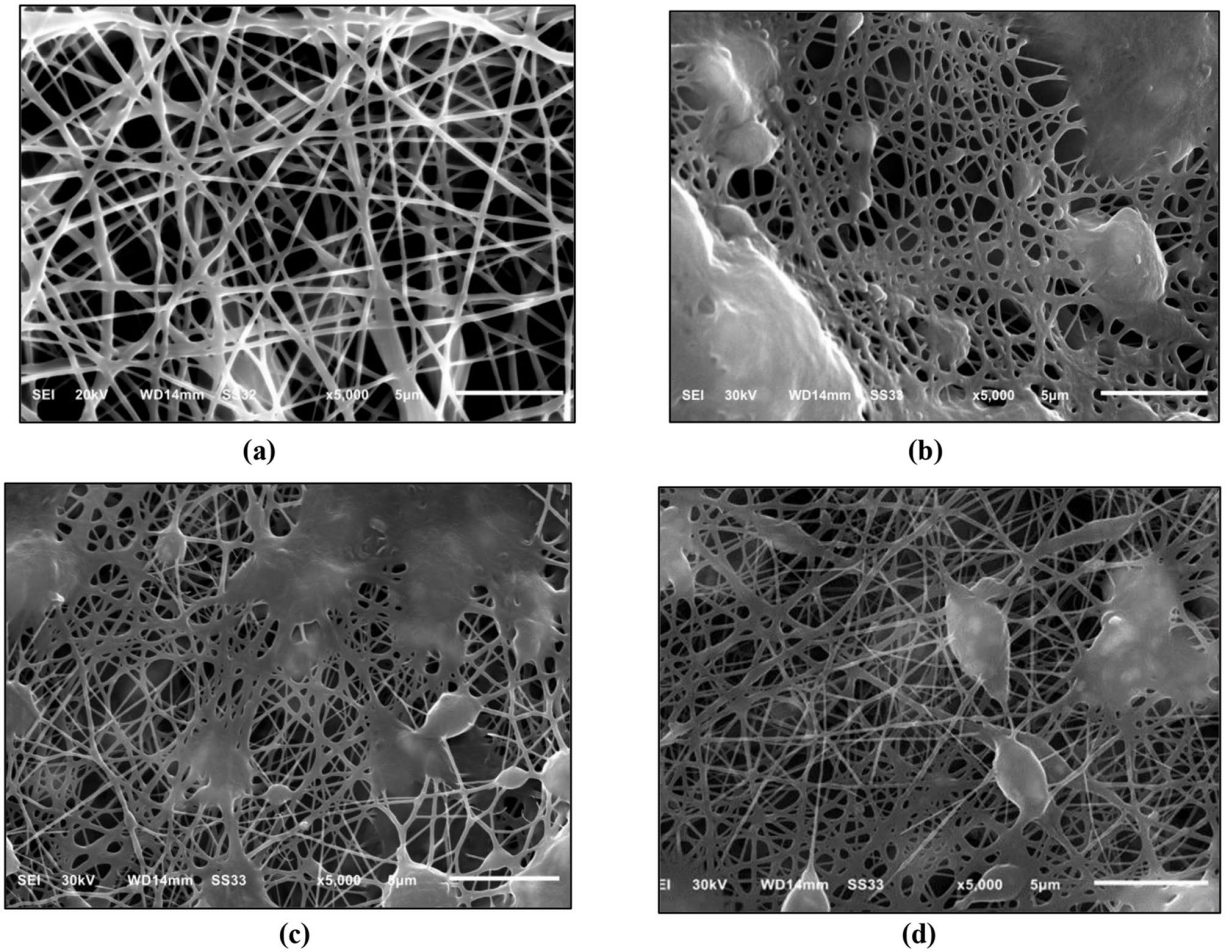
SEM images of randomly oriented TPU-based membranes, illustrating the effect of CuO NPs incorporation: (**a**) pure TPU, (**b**) TPU with 1 wt% CuO NPs, (**c**) TPU with 2 wt% CuO NPs, and (**d**) TPU with 3 wt% CuO NPs

SEM qualitative analysis was implemented to assess the morphology of the fabricated nanofibrous mats. Figures 2 (a–d) present SEM micrographs of electrospun membranes. In Fig. 2 (a), pure TPU exhibits a randomly oriented, three-dimensional network of smooth fibers with variable diameters and no bead formation. Figures 2 (b–d) show TPU/CuO mats containing 5 wt% olive oil and CuO NPs at 1, 2, and 3 wt%, respectively. Inclusion of olive oil and CuO NPs induces localized beading and partial fusion of the fiber network. As reported by Amna et al. [16], the combination of 5 wt% olive oil and 3 wt% CuO NPs produce nanofibers with an optimally fused, beaded morphology and enhanced mechanical strength, yielding superior biomedical performance compared to other formulations.

Previous studies have shown that incorporating olive oil into polymer matrices alters interchain interactions in several ways. Acting as a plasticizer, olive oil reduces intermolecular forces between polymer chains, increasing flexibility and decreasing rigidity, which manifests as lower tensile strength and higher elongation at break. Limited compatibility between olive oil and the polymer can lead to phase separation, forming distinct oil-rich and polymer-rich domains that serve as stress concentrators and introduce structural heterogeneities. Furthermore, olive oil enhances chain mobility, allowing polymer segments to slide past one another more readily. This change in molecular dynamics gives rise to fibers with modified mechanical properties and unique surface morphologies. Together, these effects produce fibers that are less rigid, more compliant, and exhibit varied structural characteristics [41–43].

**Fig. 3:**
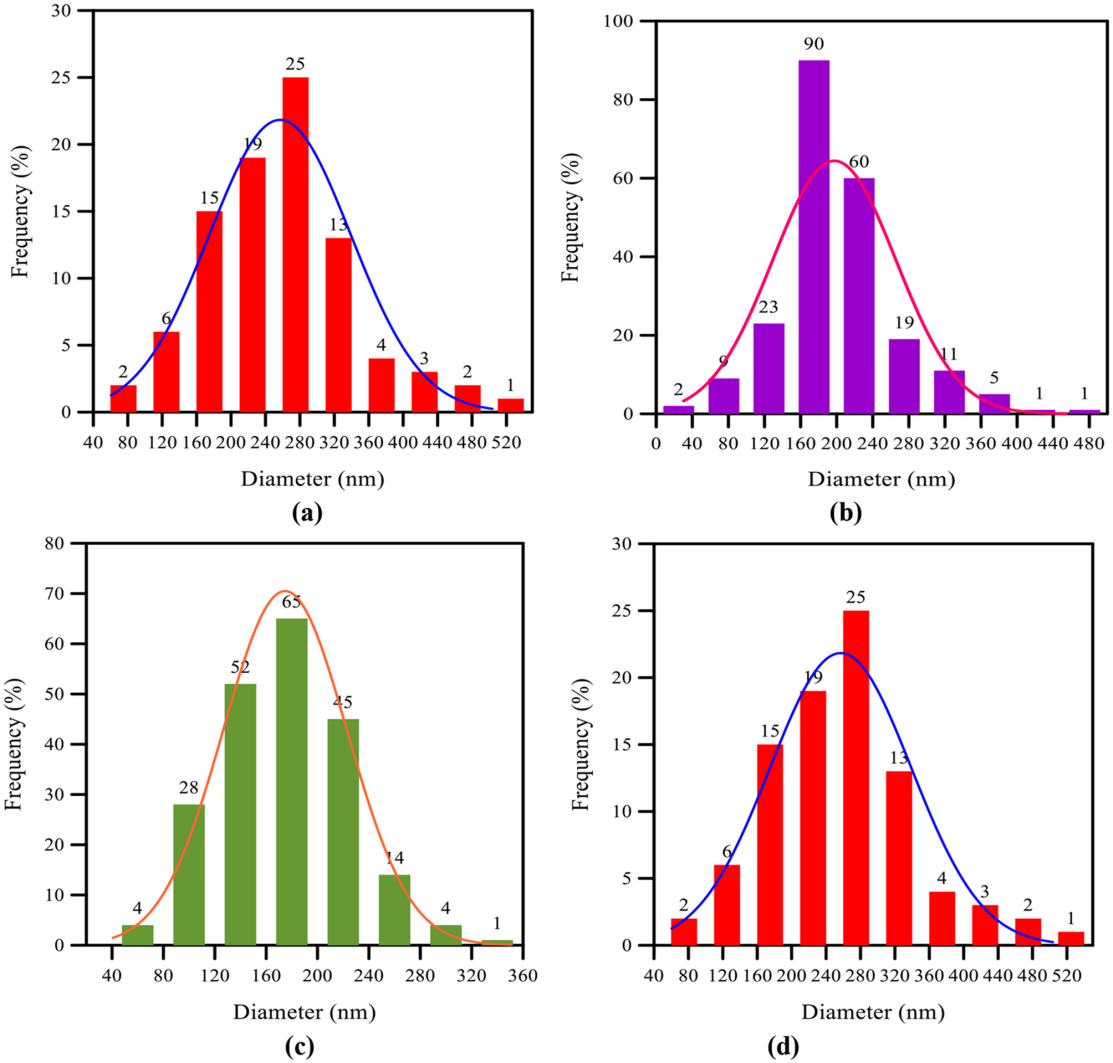
Distribution of fiber diameter for randomly oriented membranes: (**a**) pure TPU, (**b**) TPU with 1 wt% CuO NPs, (**c**) TPU with 2 wt% CuO NPs, and (**d**) TPU with 3 wt% CuO NPs

Figures 3 (a–d) illustrate the diameter distributions of randomly oriented electrospun fibers for pure TPU and TPU/CuO mats. Fiber diameters were measured in Image-J by sampling approximately 200 fibers per scaffold. Pure TPU exhibited an average diameter of 241 nm, which decreased to 197 nm, 174 nm, and 143 nm for mats containing 1, 2, and 3 wt% CuO NPs, respectively.

Previous studies have shown that incorporating CuO nanoparticles into the electrospinning solution reduces fiber diameter through multiple, complementary mechanisms. By increasing the solution’s electrical conductivity, CuO nanoparticles generate stronger electrostatic forces that stretch polymer jets into finer fibers. Simultaneously, they modulate the viscosity and surface tension of the polymer solution, facilitating jet elongation and enabling the formation of thinner fibers. In addition, these nanoparticles promote improved alignment of polymer chains during spinning, resulting in more uniform and narrower fibers. At the molecular level, interactions between CuO and the polymer matrix enhance polymer distribution throughout the jet, further contributing to reduced fiber diameters [44–46]. For instance, Khan et al. [44] have shown that adjusting the CuO nanoparticle content enables precise control over both fiber diameter and surface morphology.

**Fig. 4:**
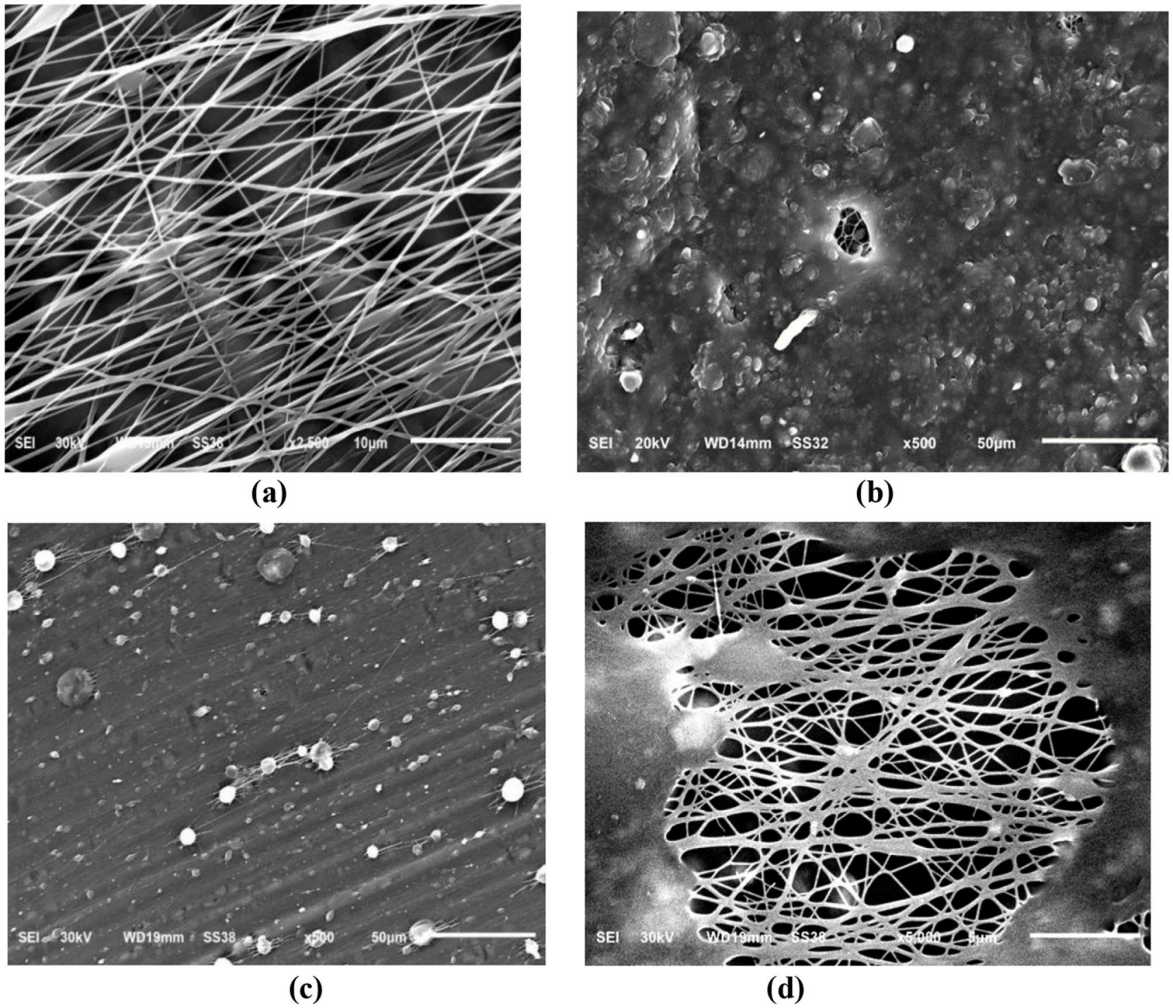
SEM images of aligned TPU-based membranes, illustrating the effect of CuO NPs incorporation: (**a**) pure TPU, (**b**) TPU with 1 wt% CuO NPs, (**c**) TPU with 2 wt% CuO NPs, and (**d**) TPU with 3 wt% CuO NPs

Figures 4 (a–d) present SEM images of unidirectionally aligned membranes of pure TPU and TPU/CuO composites. In the pure TPU samples, Fig. 4 (a), fibers align uniformly, exhibiting only minor angular deviations. The TPU/CuO mats shown in Fig. 4 (b) and (c) exhibit poorly defined, blurred fiber interfaces, while the mat containing 3 wt% CuO NPs exhibit fibers with partial fusion and clusters that disrupt alignment and induce localized deformation, as shown in Fig. 4 (d). All samples were produced under identical electrospinning conditions.

**Fig. 5:**
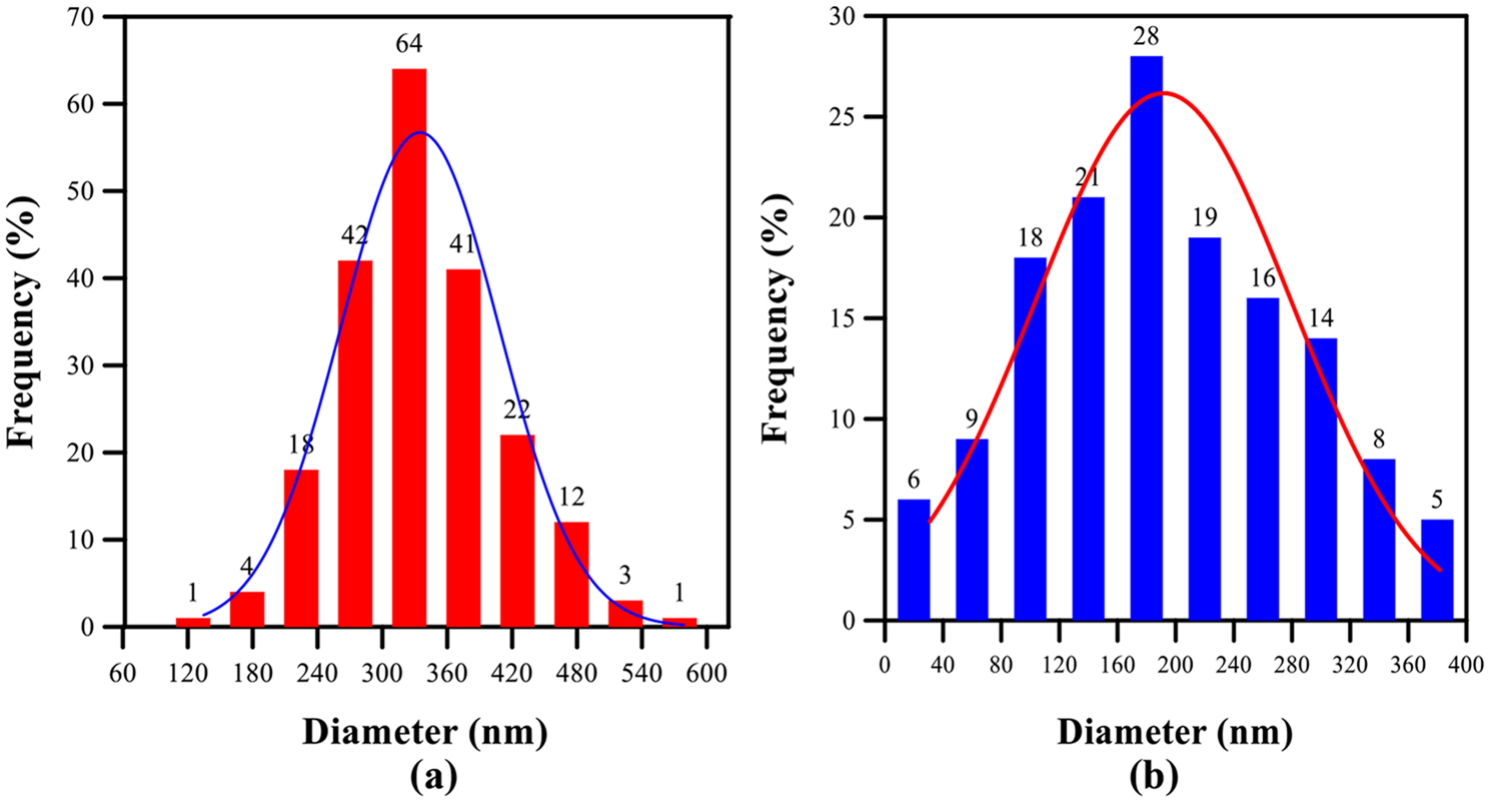
Distribution of fiber diameter for aligned membranes. (**a**) Pure TPU (**b**) TPU with 3 wt% CuO NPs

Kim et al. [47] demonstrated that collector design critically influences the morphology and physical properties of electrospun fibers, while the degree of charge dissipation during deposition dictates fiber density per unit area and overall alignment. Consistent with Amna et al. [16], our TPU/CuO hybrid mat containing 3 wt% CuO NPs and 5 wt% olive oil produced nanofibers with a beaded, fused morphology. Figures 5 (a) and (b) show the diameter distributions of aligned fibers in pure TPU and TPU/CuO scaffolds, respectively, based on measurements of approximately 200 fibers per sample using the method described above. Incorporating CuO nanoparticles reduced the mean fiber diameter from 334 nm in pure TPU mats to 191 nm in TPU/CuO samples, whereas mats with 1 and 2 wt% CuO NPs exhibited poorly defined interfaces (Fig. 4b, c), rendering diameter measurements unreliable.

When comparing pure TPU mats in random versus aligned orientations, the randomly oriented samples displayed a three-dimensional network of smooth, bead-free fibers with variable diameters averaging 241 nm. In contrast, the aligned mats featured fibers predominantly oriented along `0° with minimal angular deviation and a larger average diameter of 334 nm. Consequently, the average fiber diameter in the randomly oriented mat was approximately 28% thinner than in the aligned configuration.

Additionally, both randomly oriented and aligned TPU/CuO nanofiber mats displayed pronounced beading and partial fiber fusion. In the random mats containing 3 wt% CuO nanoparticles, the average fiber diameter was 143 nm, whereas the aligned mats measured 191 nm. Consequently, the randomly oriented fibers were 25% thinner than the aligned ones, a reduction that correlated with enhanced biological activity. Kanani et al. [48] have shown that electrospun fibers with smaller diameters possess higher specific surface areas and reduced pore sizes, features that are particularly beneficial for wound-healing applications.

### Energy dispersive analysis of X-rays (EDX)

Energy-dispersive X-ray spectroscopy (EDX) is a powerful tool for elemental analysis and chemical characterization. In this technique, a sample is irradiated with high-energy X-rays, prompting atoms within the material to emit characteristic secondary X-rays. By detecting and analyzing these emitted signals, EDX can accurately identify and quantify the elements present in a sample [49].

**Fig. 6:**
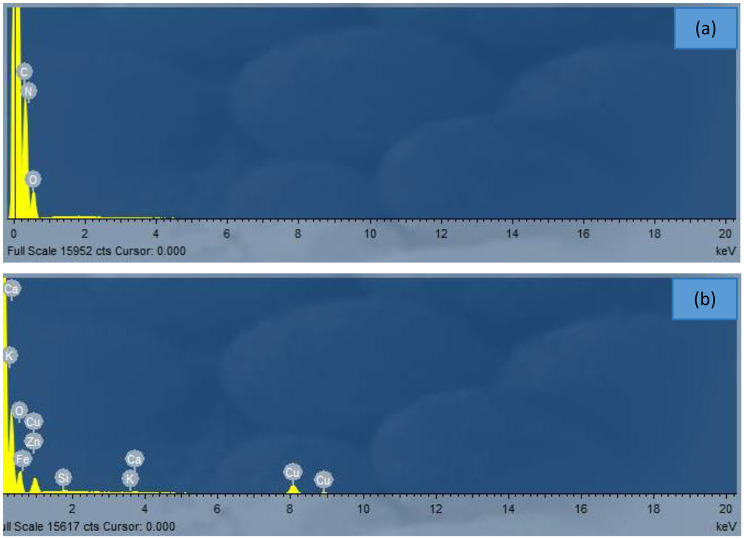
EDX spectral analysis of TPU membranes, illustrating the elemental composition of (**a**) pure TPU and (**b**) TPU/CuO composites

Figures 6 (a) and (b), along with Table 1, display the EDX profiles and elemental compositions of the TPU and TPU/CuO mats, respectively. The EDX spectroscopy of pure TPU indicates the presence of oxygen (O), nitrogen (N), and carbon (C), which are the main components of pure TPU. Previous literature has documented that the chemical composition of TPU consists of carbon as the primary element in the polymer backbone, nitrogen typically coming from urethane groups, and oxygen from ester or ether groups within the polymer structure [50, 51]. On the other hand, the EDX spectroscopy of the TPU/CuO mat confirms the presence of copper (Cu) and oxygen (O) elements, which constitute the CuO nanoparticles. Additionally, the presence of calcium (Ca), potassium (K), zinc (Zn), and iron (Fe) suggests the existence of olive oil. These findings align with similar studies conducted by other researchers [52, 53].

**Table 1:** Elemental composition data from energy dispersive X-ray (EDX) analysis for pure TPU and TPU/CuO Mats

Element	Pure TPU		TPU/CuO	
	Weight%	Atomic %	Weight%	Atomic %
O (Oxygen)	23.67	20.08	52.22	80.35
C (Carbon)	36.97	41.78	---	---
N (Nitrogen (	39.36	38.14	---	---
K (Potassium)	---	---	0.50	0.31
Ca (calcium)	---	---	1.16	0.71
Fe (Iron)	---	---	0.33	0.15
Cu (Copper)	---	---	42.55	16.49
Zn (Zinc)	---	---	1.69	0.64
Si (Silicon)	---	---	1.54	1.35

### Fourier-transform infrared (FT-IR) analysis

The structural variations resulting from covalent bond interactions between the functional groups were studied utilizing the Fourier-transform infrared (FT-IR) characterization technique for verifying the formation of the TPU/CuO hybrid mat via electrospinning [54].

**Fig. 7:**
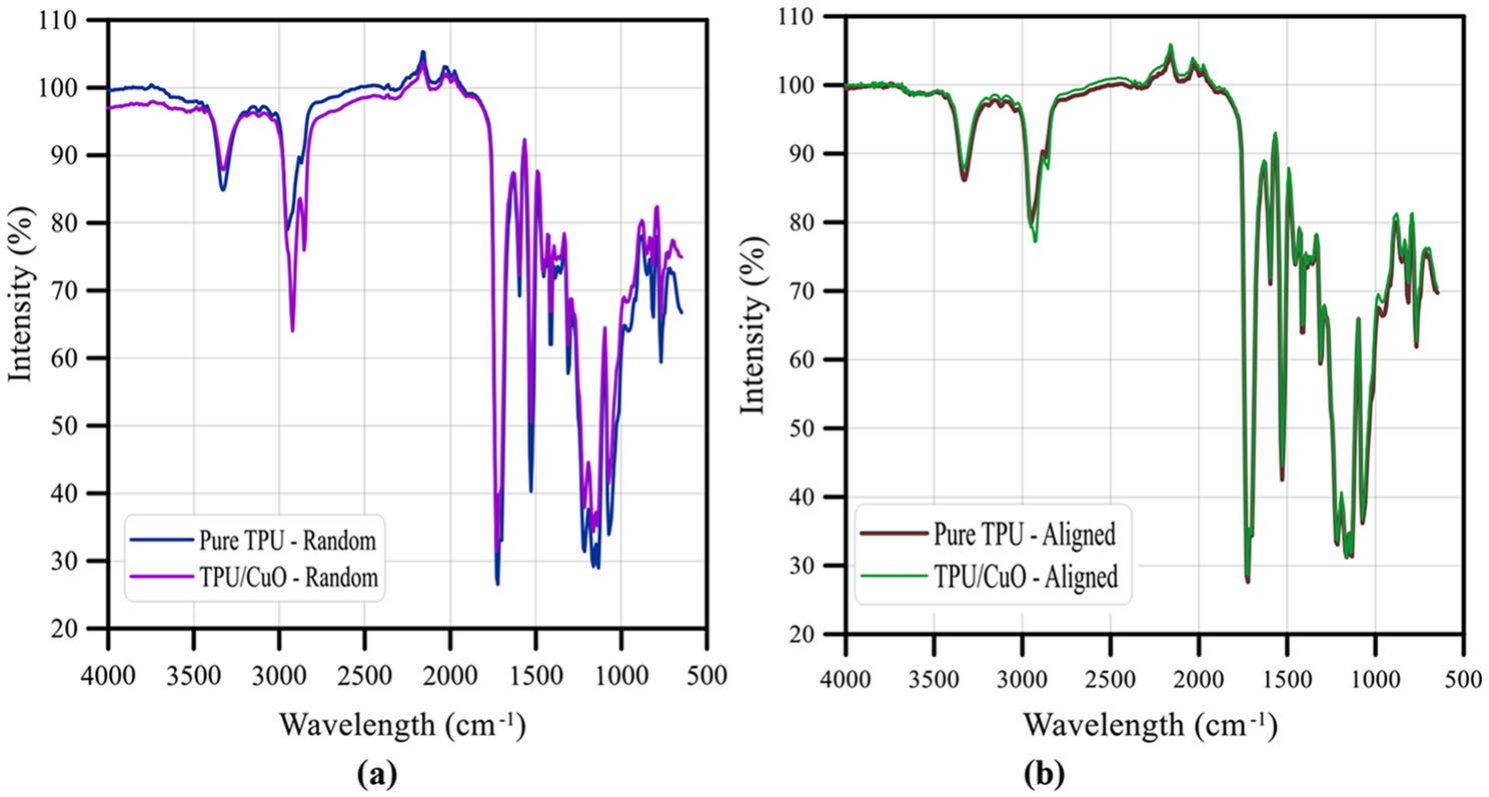
Spectra of Fourier-transform infrared analysis for TPU and TPU/CuO (**a**) randomly oriented membranes, and (**b**) aligned membranes

Figures 7 (a) and (b) show the FT-IR spectra of TPU and TPU/CuO mats for both oriented structures of the membranes, respectively. The pure TPU membrane’s spectra show peaks at 3422 cm^− 1^ (N-H), 2924 cm^− 1^ (C - H), 1169 cm^− 1^ (C = O),1570 cm^− 1^ (C = C), 1233 cm^− 1^ (C - C), and 1071 cm^− 1^ (C - O). The CuO NPS spectra show peaks at 2933 and 3432 cm^− 1^ (O–H),1410 and 1564 cm^− 1^ (C = O), and 1639 cm^− 1^ (Cu–O) where the occurrence of banding at 523 cm^− 1^ and 1011 cm^− 1^ signifies that the Cu-O bond has multiple modes of bending vibration, and olive oil show peaks at 3007 and 1654 cm^− 1^ for C = H and C = C, respectively.

Shaker et al. [55] recorded that the TPU membrane’s spectra have peaks for (C-O) at 1070 cm^− 1^, (C-C) at 1232 cm^− 1^, (C = C) at 1569 cm^− 1^, (C = O) at 1670 cm^− 1^, (C-H) at 2923 cm^− 1^, and (N-H) at 3423 cm^− 1^. Additionally, previous literature reported that FT-IR analysis to characterize the surface nature of the CuO nanoparticles has peaks at 1410 and 1564 cm^− 1^ corresponding to C = O stretching of carboxylate ion bond to the CuO nanoparticles. The bands that occur at 3432 cm^− 1^ and 2933 cm^− 1^ are related to the asymmetrical and symmetrical vibrations of the stretching (O-H) bond, respectively. Peaks at 523 cm^− 1^ and 1011 cm^− 1^ indicate different modes of bending vibration of the (Cu-O) bond, while the peak at 1639 cm^− 1^ indicates stretching vibration of the (Cu-O) bond of copper (II) oxide [56, 57]. Zarghami et al. [43], recorded that olive oil’s C-H and C = C peaks were observed at 3007 and 1654 cm^− 1^, respectively.

In comparing the FT-IR spectra of the pure TPU and TPU/CuO mats for both orientations, it was observed that all materials displayed the same peaking behavior at varied amplitudes. Whereas the amplitude of these peaks increases with the polymerization chain length. Additionally, the existence of peaks for (C = C), (C-H), and (Cu–O) bonds is according with the addition of olive oil and CuO NPs. These identical peaks in the TPU/CuO mat structure indicated that CuO NPS and olive oil were embedded within the TPU/CuO composite nanofiber.

### Antimicrobial activity of randomly oriented scaffolds

Considering the distinctive properties of nanoparticles and recent advances in nanotechnology, researchers have engineered novel compounds with significant biomedical and biotechnological potential. Table 2; Fig. 1S (supplementary) present a summary of the antimicrobial performance of randomly oriented TPU and TPU/CuO scaffolds. The activity of CuO NPs incorporated into TPU fibers was evaluated against a spectrum of pathogens, Gram-negative bacteria (*K. pneumonia* and E. *coli*), Gram-positive bacteria (*Staphylococcus aureus* and *Bacillus subtilis*), and the filamentous fungus (*Aspergillus brasiliensis*) using a disc-diffusion bioassay.

**Table 2:** Determination of the antimicrobial activity of the randomly oriented scaffolds by disc-diffusion bioassay

Microbial strains (ATCC)	Diameter of inhibition zone (mm)			
	Pure TPU	TPU/CuO (1 wt% CuO NPs) (mm)	TPU/CuO (2 wt% CuO NPs)	TPU/CuO (3 wt% CuO NPs)
*B. subtilis*	0.0 ± 10.0 **d**	1.0 ± 15.0 **c**	23.66 ± 1.52 **b**	28.0 ± 1.0 **a**
*S. aureus*	0.0 ± 10.0 **d**	± 18.0 1.0 **c**	± 25.66 1.52 **b**	± 33.0 1.0 **a**
*E. coli*	0.0 ± 10.0 **a**	0.0 ± 10.0 **a**	0.0 ± 10.0 **a**	0.0 ± 10.0 **a**
*K. pneumoniae*	0.0 ± 10.0 **c**	0.0 ± 10.0 **c**	0.57 ± 12.61 **b**	1.68 ± 19.46 **a**
*A. brasiliensis*	0.0 ± 10.0 **a**	0.0 ± 10.0 **a**	0.0 ± 10.0 **a**	0.0 ± 10.0 **a**

Data represented by Mean ± SD (*n* = 3). Different lower-case letters in the same row are significantly different by post hoc Tukey’s test at *p* < 0.05; values in the same row with the same letter are not significantly different

Copper oxide nanoparticles demonstrated a concentration-dependent antibacterial effect against a range of pathogens, in agreement with earlier reports on their antibacterial efficacy [16, 58–61]. The TPU/CuO mat containing 3 wt% CuO NPs, representing the highest nanoparticle loading, demonstrated the most pronounced antibacterial activity. It has produced the largest inhibition zones 28.0 ± 1.0 mm for *Bacillus subtilis*, 33.0 ± 1.0 mm for *Staphylococcus aureus*, and 19.46 ± 1.68 mm for *Klebsiella pneumoniae*, while the pristine TPU mat (TPU Pure) showed no antimicrobial activity as demonstrated in Table 2. A similar trend, albeit with slightly smaller zones, was observed for the TPU/CuO with 2 wt% of CuO NPs sample, Figs. 1S and 2S (Supplementary). Khan et al. [46] likewise reported that CuO NPs inhibited various bacterial species with zones measuring 20–32 mm. Furthermore, copper in its ionic form (Cu2+) is approved by the U.S. Environmental Protection Agency as a safe and effective antimicrobial agent.

**Fig. 8:**
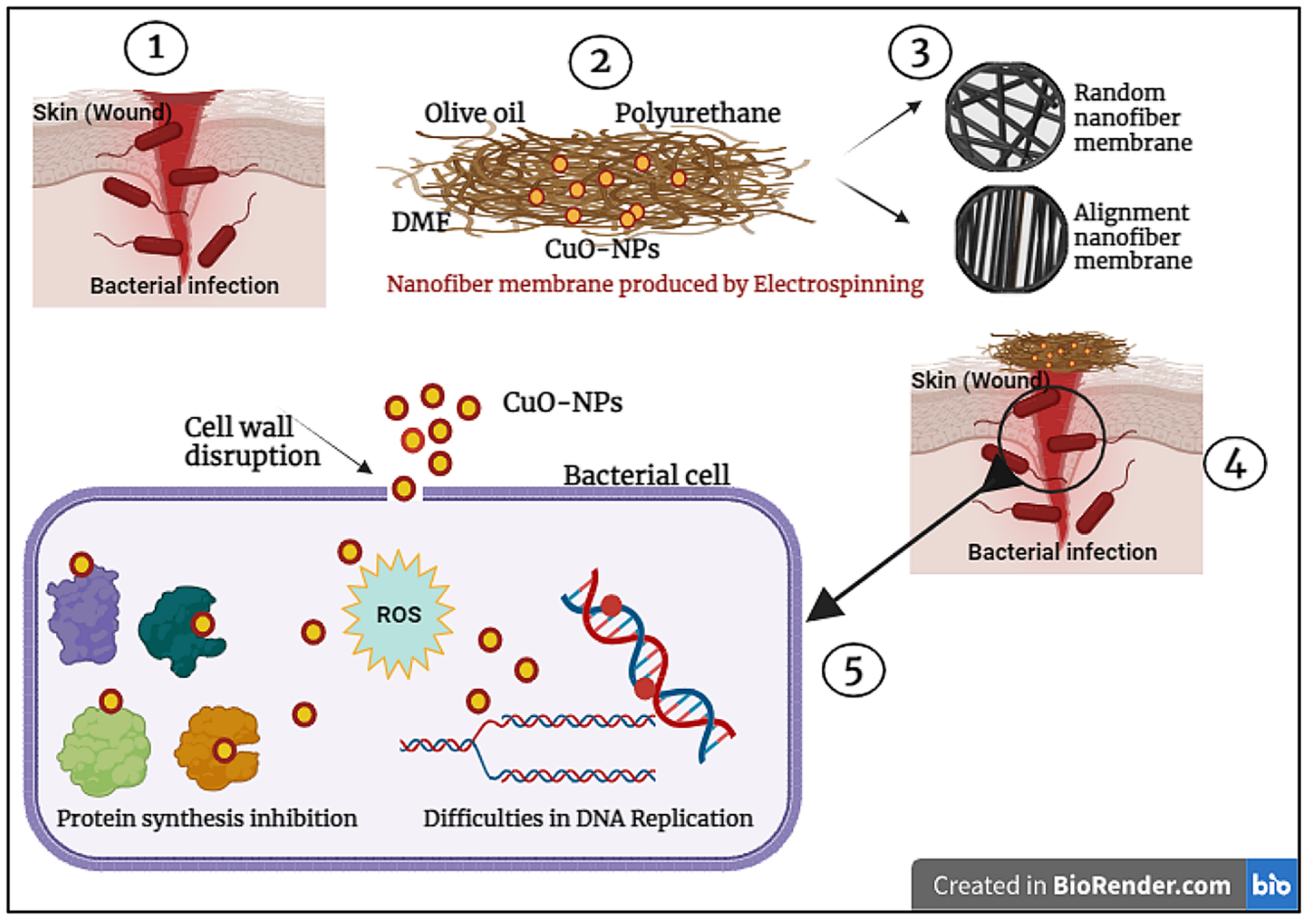
Proposed schematic illustration of the antimicrobial mechanisms of action of TPU/CuO nanofibers against bacterial pathogens

Figure 8 illustrates that CuO nanoparticles directly engage with microbial cells, mechanically disrupting and penetrating their membranes to exert potent inhibitory effects against infection [62]. Furthermore, exposure to CuO NPs triggers the generation of reactive oxygen species such as hydrogen peroxide, superoxide anions, and hydroxyl radicals, which induce DNA strand breaks, suppress mRNA and protein synthesis, and culminate in cell death [63]. The pronounced antimicrobial activity can also be attributed to the nanoparticles’ small size and spherical morphology, which facilitate efficient cellular uptake. Indeed, numerous studies have shown that reducing particle dimensions enhances antimicrobial efficacy [64, 65]. Together, membrane disruption, oxidative stress, and impaired nutrient absorption underpin the robust inhibitory action of CuO NPs on pathogenic microorganisms [66].

### Antimicrobial activity of aligned oriented scaffolds

Table 3; Fig. 3S (Supplementary) summarize the antimicrobial activity of aligned TPU and TPU/CuO scaffolds. The antibacterial efficacy of CuO nanoparticles incorporated into aligned TPU fibers was evaluated against Gram-positive bacteria (*S. aureus* and *B. subtilis*), using a disc-diffusion bioassay.

**Table 3:** Determining the antimicrobial activity of aligned oriented pure TPU and TPU/CuO scaffolds by disc-diffusion bioassay

Microbial strains (ATCC)	Diameter of inhibition zone (mm)			
	Pure TPU	TPU/CuO (1 wt% CuO NPs)	TPU/CuO (2 wt% CuO NPs)	TPU/CuO (3 wt% CuO NPs)
*B. subtilis*	0.0 ± 10.0 **c**	0.45 ± 14.7 **b**	15.30 ± 0.95 **b**	27.63 ± 1.92 **a**
*S. aureus*	0.0 ± 10.0 **c**	0.0 ± 10.0 **c**	± 25.33 1.15 **b**	± 31.7 1.47 **a**

Data represented by Mean ± SD (*n* = 3). Different lower-case letters in the same row are significantly different by post hoc Tukey’s test at *p* < 0.05; values in the same row with the same letter are not significantly different

Aligned TPU/CuO membranes containing the highest CuO nanoparticle loading (3 wt%) exhibited the greatest antimicrobial efficacy, with inhibition zones measuring 27.63 ± 1.92 mm *against B. subtilis* and 31.7 ± 1.47 mm against S. aureus. In contrast, the aligned pure TPU scaffold, which lacked CuO nanoparticles, showed no antimicrobial activity as illustrated in Table 3; Figs. 3S and 4S (Supplementary).

A comparative evaluation of randomly oriented and aligned TPU/CuO membranes shows that the randomly oriented samples inhibit Gram-positive bacteria (*B. subtilis and S. aureus*), Gram-negative bacteria (*E. coli and K. pneumoniae*), and the fungi (*A. brasiliensis*). In contrast, the aligned-oriented membranes exhibit antimicrobial activity exclusively against the Gram-positive strains (*B. subtilis and S. aureus*).

Additionally, when comparing the antimicrobial activity of randomly oriented pure TPU with that of TPU/CuO membranes, a significant increase in activity is observed. For example, against *B. subtilis*, the activity rose from 10 mm for pure TPU to 28 mm for TPU/CuO with 3 wt% of CuO NPs, showing 180% improvement. Similarly, against *S. aureus*, the activity increased from 10 mm for pure TPU to 33.0 mm for TPU/CuO with 3 wt% of CuO NPs, showing a 230% improvement. Furthermore, the activity against *K. pneumoniae* increased from 10 mm for pure TPU to 19.46 mm for TPU/CuO with 3 wt% of CuO, demonstrating a 94.6% improvement. This activity decreased with lower CuO NP concentrations, reaching 18.0 ± 1.0 mm and 15.0 ± 1.0 mm, respectively.

**Fig. 9:**
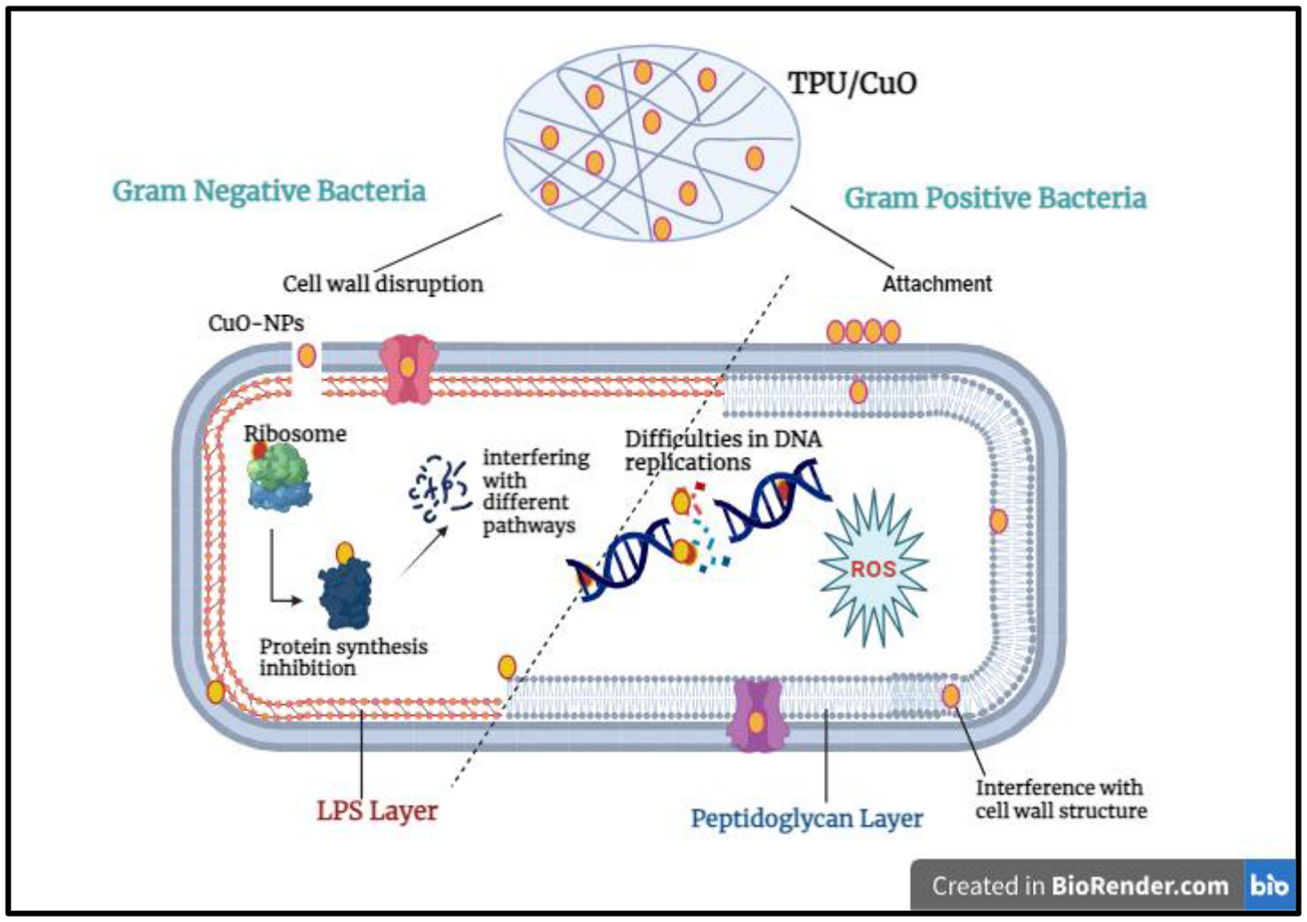
Conceptual schematic illustrating the proposed interaction mechanisms of CuO nanoparticles with Gram-positive and Gram-negative bacteria based on differences in cell wall structure

In contrast, TPU/CuO with 1 wt% CuO nanoparticles showed clear antimicrobial effects against Gram-positive strains (S. aureus and B. subtilis), while Gram-negative strains displayed no detectable activity at this concentration. Gram-negative bacteria, such as *E. coli*, have an exterior lipopolysaccharide (LPS) layer on their surface that limits the entry of chemicals [67]. Figure 9, which may explain their lower susceptibility to NPs. However, higher doses of antibacterial drugs are needed to have the same effect on Gram-positive bacteria [68].

In comparison of the antimicrobial activity of the aligned oriented pure TPU with TPU/CuO membranes, the activity increased from 10 mm for pure TPU to 27.63 mm for TPU/CuO with 3 wt% of CuO NPs membrane against *B. subtilis*, which showed 176.3% improvements. Additionally, the activity increased from 10 mm for pure TPU to 31.7 mm for TPU/CuO (3 wt% CuO NPs) membrane against *S. aureus*, which showed a 217% improvement.

In addition to its wide-ranging antibacterial properties, copper is essential for several physiological and metabolic functions, including angiogenesis, endothelial growth stimulation, and extracellular skin protein stability [17]. Similarly, there is very little unpleasant skin sensitivity to copper [69]. Conclusively, an organic/inorganic hybrid micro nanofibrous dressing was created by simple electrospinning, taking into account the ability of nanofibrous polyurethane (PU) membranes to stimulate the growth of epithelial [70], and the previously indicated applications of copper and olive oil. We think these nanofibers promote rapid wound healing and lower the risk of infection.

In general conclusion the antimicrobial performance of the electrospun scaffolds was quantitatively evaluated using the disc-diffusion assay, and the results clearly demonstrate statistically significant differences among the tested formulations. As shown in Tables 2 and 3, random-oriented TPU/CuO scaffolds exhibited significantly larger inhibition zones than their aligned counterparts against *B. subtilis* and *S. aureus* at CuO loadings of 2 and 3 wt% (*p* < 0.05). In contrast, pure TPU and low CuO concentrations showed no significant antimicrobial effect, confirming that the observed activity originates from the incorporated CuO nanoparticles. While these results statistically support the superior antimicrobial performance of the randomly oriented mats, the underlying reasons for this enhancement are interpreted qualitatively. The smaller fiber diameter and more disordered architecture of the random mats are likely to increase the effective surface area and promote more intimate contact between the scaffold surface and microbial cells, which may facilitate improved antimicrobial interactions [71, 72].

### Hemostatic performance

**Fig. 10:**
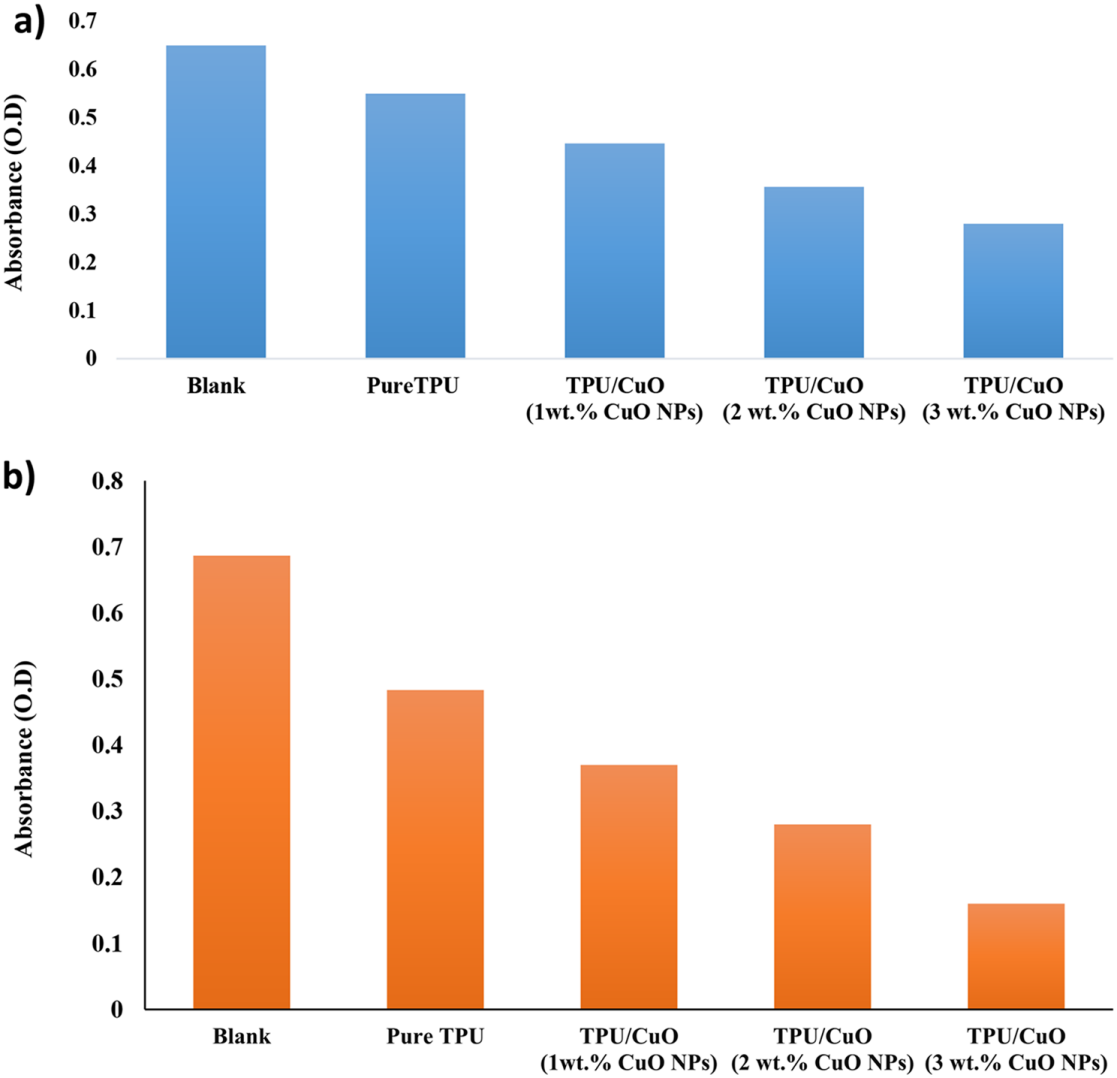
Corresponding absorbance of hemoglobin from hemolyzed, uncoagulated RBCs for (**a**) the randomly oriented and (**b**) the aligned membranes

The whole blood clotting analysis was carried out to assess the hemostatic capabilities of the randomly oriented TPU/CuO and aligned oriented TPU/CuO membranes. Figure 10 shows the comparable absorbance of hemoglobin generated from hemolyzed uncoagulated red blood cells. A higher hemoglobin solution absorbance value indicates a slower rate of clotting. According to the data, within 15 min, the TPU/CuO of the aligned-oriented mat and the randomly oriented membrane have a better hemostatic effect than the membranes.\.

The distinct physicochemical features of TPU nanofiber are associated with its hemostatic action mechanism. Hemostasis depends on materials having a high surface area and a porous structure [39]. High surface area encourages erythrocyte and platelet adherence, whereas the porous structure guarantees rapid absorption of wound exudates and substantially inhibits erythrocyte and platelet escape [73].

### In vitro cytotoxicity of the hybrid mat

**Fig. 11:**
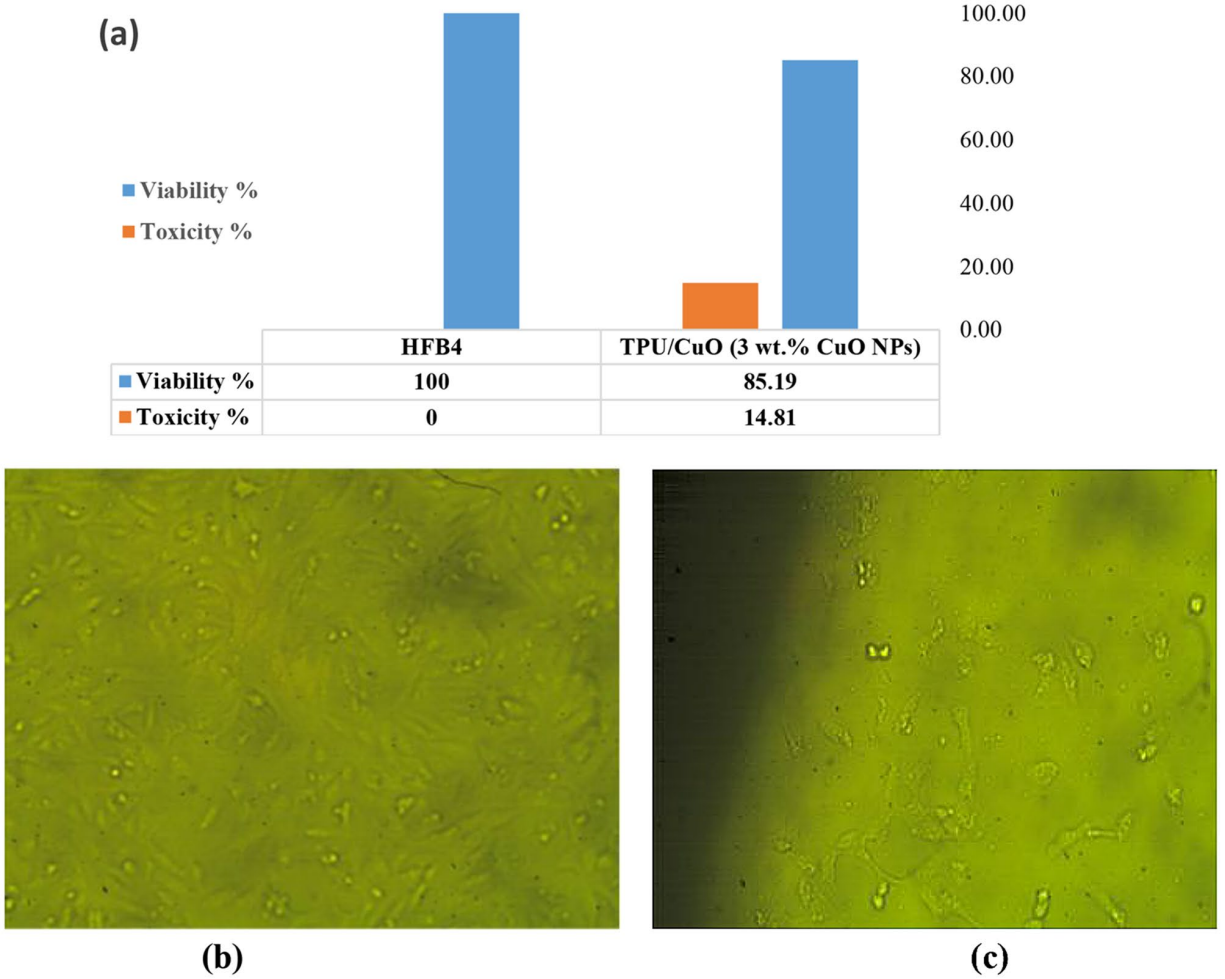
Cytocompatibility test results: (**a**) the viability of HFB4 on the randomly oriented TPU/CuO membrane, (**b**) normal skin cells (HFB4) imaged by a light inverted microscope, and (**c**) the effect of the randomly oriented TPU/CuO membrane on normal skin cells (HFB4) imaged by a light inverted microscope

**Fig. 12:**
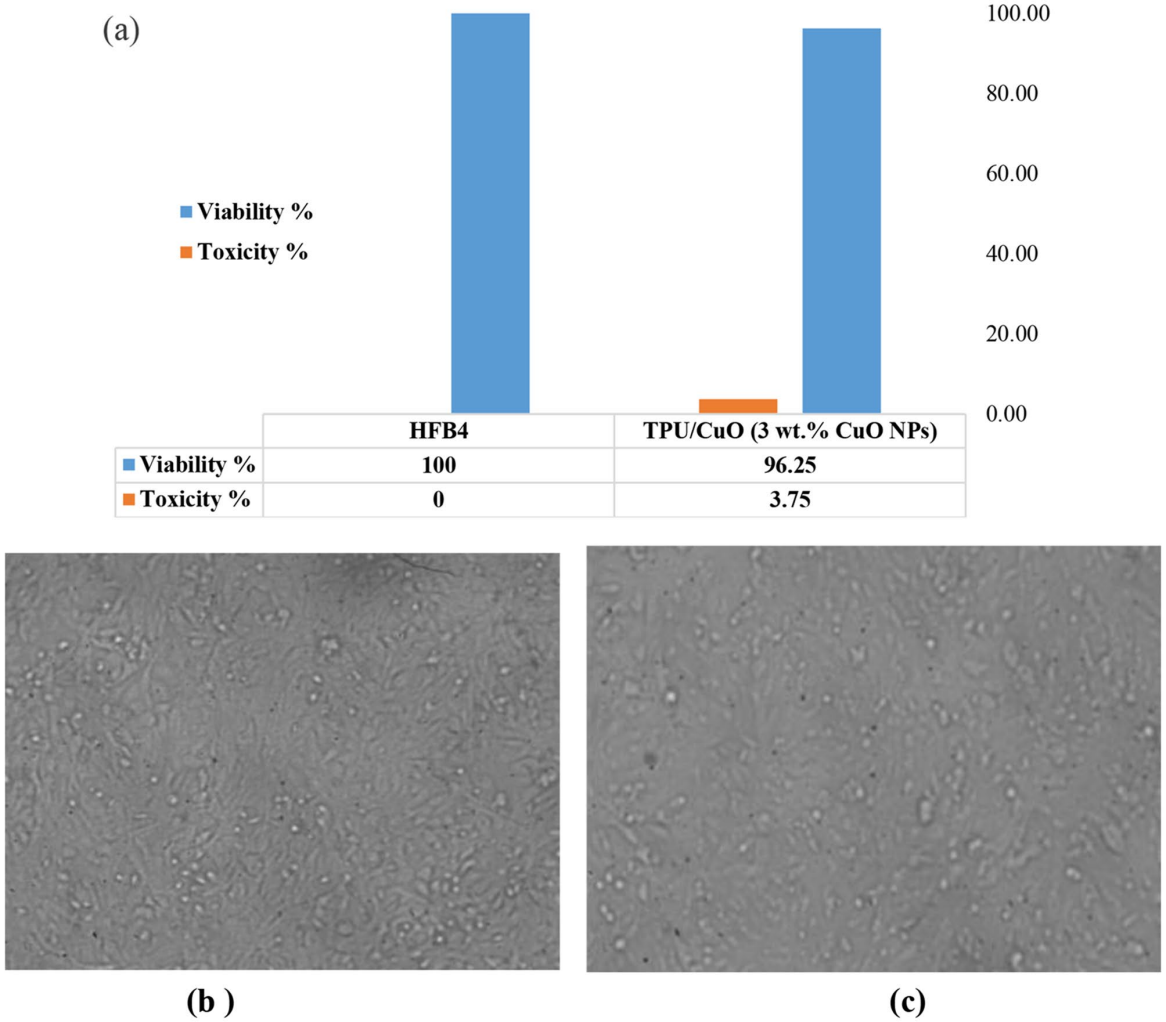
Cytocompatibility test results: (**a**) the viability of HFB4 on the aligned oriented TPU/CuO membrane, (**b**) normal skin cells (HFB4) imaged by a light inverted microscope, and (**c**) the effect of the aligned TPU/CuO membrane on normal skin cells (HFB4) imaged by a light inverted microscope

The objective of this study is to evaluate the safety and potential of TPU/CuO membranes for use in skin wound healing, specifically targeting the prevention of microbial infections. To determine the biocompatibility of the membrane, an in vitro cytotoxicity assessment was conducted using the MTT assay on HFB4 cells, a normal cell line [74]. The results, presented in Figs. 11 and 12, provide insight into the membrane’s cellular response and its suitability for biomedical applications.

**Fig. 13:**
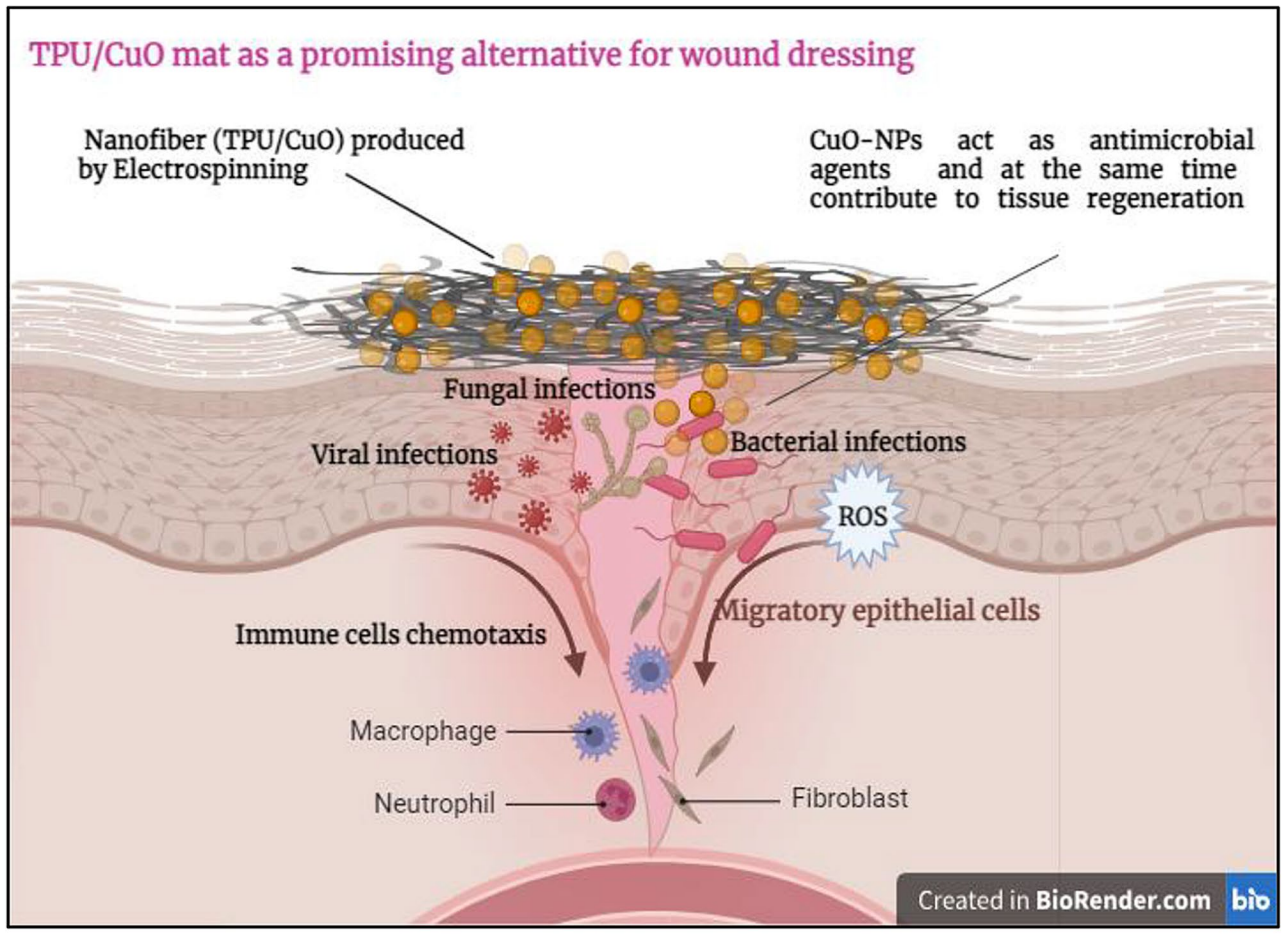
Conceptual schematic diagram illustrating the proposed role of TPU/CuO nanofibrous mats as wound dressing materials for skin injury healing

In comparing the viability of a normal cell line that attaches and grows on TPU/CuO (3 wt% of CuO NPs) membranes in both orientations, the viability increased from 85.19% for TPU/CuO in randomly oriented membranes to 96.25% for TPU/CuO in aligned membranes. The aligned-oriented membranes showed a reduction in cell viability of less than 5%, with overall viability remaining well above the ISO 10993-5 cytotoxicity threshold against normal skin cells (HFB4). According to ISO 10993-5 guidelines [75], materials exhibiting cell viability values greater than 70% are considered non-cytotoxic. In this study, both randomly oriented (~ 85%) and aligned (~ 96%) TPU/CuO nanofibrous membranes demonstrated cell viabilities well above this threshold, indicating their non-cytotoxic nature. The high cell viability observed for TPU/CuO membranes further confirms their biocompatibility when evaluated against internationally accepted standards. In particular, the aligned TPU/CuO membranes exhibited cell viability values exceeding 95%, suggesting excellent cytocompatibility suitable for wound dressing applications. As a result, the TPU/CuO (3 wt% of CuO NPs) mat is regarded as biocompatible in vitro, making it a promising alternative for wound dressing. Figure 13. Li et al. [76] also reported that after five days of culturing cells, there were more live cells on aligned fibers than on random counterparts. Furthermore, Tang et al. [77] reported that the extracellular matrix contractions and cell survival of nanofibers can be enhanced by their aligned degree when compared to random.

## Conclusions

In this study, thermoplastic polyurethane nanofibrous scaffolds incorporating copper oxide nanoparticles and olive oil were successfully fabricated in both random and aligned orientations to evaluate how structural architecture and composition influence their biological performance. The findings demonstrate that fiber orientation plays a decisive role in functional behavior: randomly oriented mats provided greater surface area and more accessible contact points, which translated into enhanced antimicrobial activity and hemostatic performance. Meanwhile, aligned fibers supported excellent cytocompatibility and promoted favorable cell responses.

Among all formulations, scaffolds containing 3 wt% CuO nanoparticles exhibited the most balanced performance, delivering strong antimicrobial activity together with acceptable biocompatibility. Olive oil contributed synergistically by improving fiber formation, enhancing flexibility, and supporting cell interaction without acting as a primary antimicrobial agent. Together, these factors produced multifunctional nanofibrous membranes capable of addressing the key clinical needs of wound dressing materials.

## Supplementary Information

Below is the link to the electronic supplementary material.

Supplementary Material 1: Fig. 1 S: Antimicrobial activity against different pathogenic microorganisms by exposure to randomly oriented membranes. Lower-case letters in the same strain are significantly different by post hoc Tukey’s test at *p* < 0.05; values in the same strain with the same letter are not significantly different. Fig. 2 S: Antimicrobial activity against (a) gram-positive, (b) gram-negative, and (c) fungal strains by exposure to the randomly oriented membranes. Fig. 3 S: Antimicrobial activity against different pathogenic microorganisms by exposure to aligned pure TPU and TPU/CuO membranes. Different lower-case letters in the same strain are significantly different by post hoc Tukey’s test at *p* < 0.05; values in the same strain with the same letter are not significantly different. Fig. 4 S: Antimicrobial activity against different pathogenic microorganisms is achieved by exposure to aligned oriented pure TPU and TPU/CuO membranes using the agar disc diffusion bioassay method

## Data Availability

The data used to support the findings of this study are available from the corresponding author upon request.
