# Supplementary material for: Polyurethane nanofibrous scaffolds for wound repair: from electrospinning fabrication to biological performance evaluation
Source: BMC Biotechnol. 2026 Mar 11;26:43. doi: 10.1186/s12896-026-01114-4 (PMC13063895; doi:10.1186/s12896-026-01114-4)
Supplement: Supplementary file 1 — Supplementary Material 1: Fig. 1 S: Antimicrobial activity against different pathogenic microorganisms by exposure to randomly oriented membranes. Lower-case letters in the same strain are significantly different by post hoc Tukey’s test at p < 0.05; values in the same strain with the same letter are not significantly different. Fig. 2 S: Antimicrobial activity against (a) gram-positive, (b) gram-negative, and (c) fungal strains by exposure to the randomly oriented membranes. Fig. 3 S: Antimicrobial activity against different pathogenic microorganisms by exposure to aligned pure TPU and TPU/CuO membranes. Different lower-case letters in the same strain are significantly different by post hoc Tukey’s test at p < 0.05; values in the same strain with the same letter are not significantly different. Fig. 4 S: Antimicrobial activity against different pathogenic microorganisms is achieved by exposure to aligned oriented pure TPU and TPU/CuO membranes using the agar disc diffusion bioassay method [file 12896_2026_1114_MOESM1_ESM.docx]

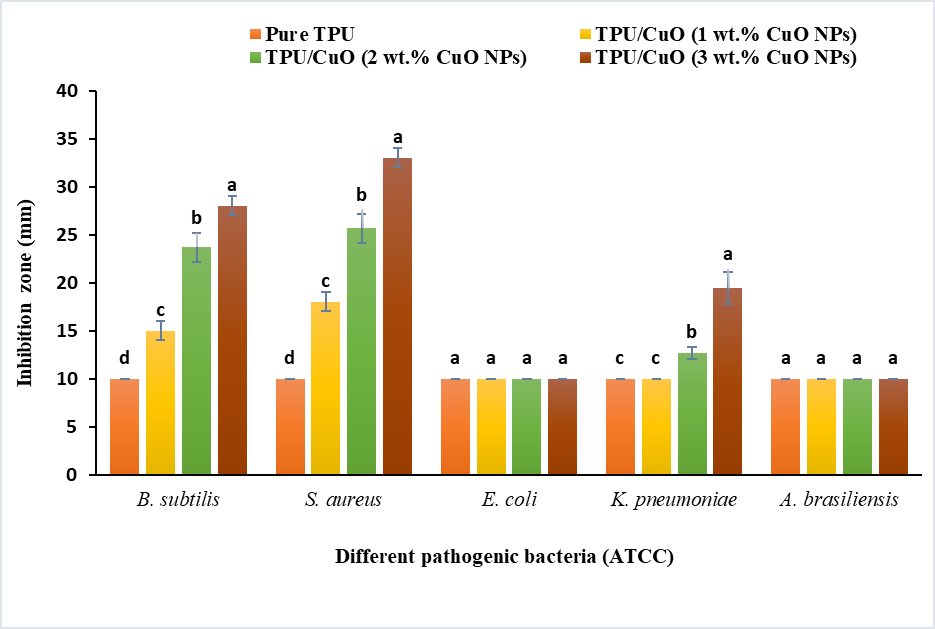


**Figure 1S.** Antimicrobial activity against different pathogenic microorganisms by exposure to randomly oriented membranes. Lower-case letters in the same strain are significantly different by post hoc Tukey's test at p < 0.05; values in the same strain with the same letter are not significantly different.


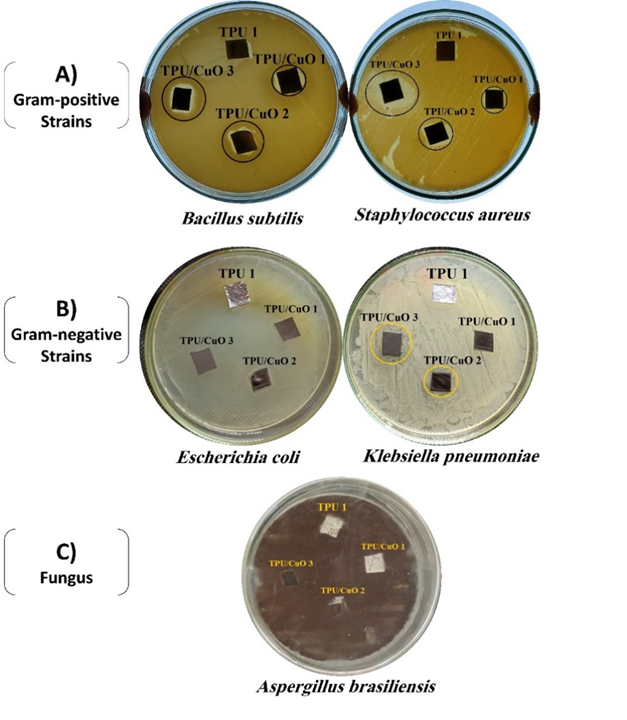


**Figure 2S**. Antimicrobial activity against (a) gram-positive, (b) gram-negative, and (c) fungal strains by exposure to the randomly oriented membranes.

**Figure 3S.** Antimicrobial activity against different pathogenic microorganisms by exposure to aligned pure TPU and TPU/CuO membranes. Different lower-case letters in the same strain are significantly different by post hoc Tukey's test at p < 0.05; values in the same strain with the same letter are not significantly different.


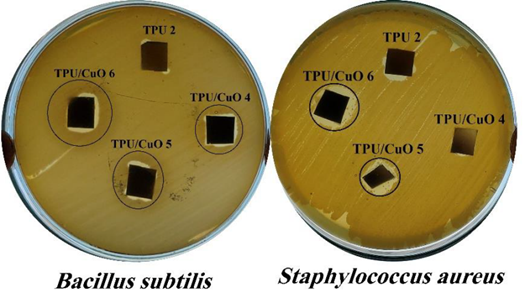


**Figure 4S**. Antimicrobial activity against different pathogenic microorganisms is achieved by exposure to aligned pure TPU and TPU/CuO membranes using the agar disc diffusion bioassay method
